# SarA based novel therapeutic candidate against *Staphylococcus aureus* associated with vascular graft infections

**DOI:** 10.3389/fmicb.2015.00416

**Published:** 2015-05-06

**Authors:** Rekha Arya, R. Ravikumar, R. S. Santhosh, S. Adline Princy

**Affiliations:** ^1^Quorum Sensing Laboratory, Centre for Research on Infectious Diseases, School of Chemical and Biotechnology, SASTRA UniversityThanjavur, India; ^2^Department of Chemistry, SASTRA UniversityThanjavur, India; ^3^Genetic Engineering Laboratory, Centre for Research on Infectious Diseases, School of Chemical and Biotechnology, SASTRA UniversityThanjavur, India

**Keywords:** *Staphylococcus aureus*, multi drug resistance, SarA, quorum sensing, molecular docking, virulence gene expression, vascular graft associated infection

## Abstract

*Staphylococcus aureus* is a common pathogen seen in prosthetic vascular graft, leading to high morbidity and mortality. The virulence genes for severity of infections are under the control of global regulators. Staphylococcal accessory regulator A (SarA) a known master controller of biofilm formation is an attractive target for the drug development. A structure based screening of lead compounds was employed for the identification of novel small molecule inhibitors targeted to interact to the DNA binding domain of the transcriptional activator, SarA and hinder its response over the control of genes that up-regulate the phenotype, biofilm. The top-hit SarA selective inhibitor, 4-[(2,4-diflurobenzyl)amino] cyclohexanol (SarABI) was further validated *in-vitro* for its efficacy. The SarABI was found to have MBIC_50_value of 200 μg/ml and also down-regulated the expression of the RNA effector, (*RNAIII*), Hemolysin *(hld*), and fibronectin-binding protein (*fnbA*). The anti-adherence property of SarABI on *S. aureus* invasion to the host epithelial cell lines (Hep-2) was examined where no significant attachment of *S. aureus* was observed. The SarABI inhibits the colonization of MDR *S. aureus* in animal model experiment significantly cohere to the molecular docking studies and *in vitro* experiments. So, we propose that the SarABI could be a novel substitute to overcome a higher degree of MDR *S. aureus* colonization on vascular graft.

## Introduction

*Staphylococcus aureus* is the most commonly isolated pathogen in the prosthetic vascular graft implanted in patients and uses a synchronized multiple virulence gene expression to establish infection in humans (Legout et al., 2012) and leads to organ failure, and death particularly in immunocompromised patients (Barnes and Chetter, 2012). The associated infections cause severe clinical threat because of the greater morbidity and mortality related to its opportunistic behavior (Lister and Horswill, 2014). *S. aureus* colonizes in prosthetic grafts to form a remarkable multilayer biofilm that is very difficult to treat clinically since the bacterial cells within the biofilm are resistant to the host immune response and antibiotic agents (Daghighi et al., 2013).

Various pathways have been elucidated in *S. aureus* that are linked with its pathogenicity and virulence gene expression. The RNAIII is the intracellular effector of the *agr* quorum sensing mechanism to coordinate a large number of virulence determinants including cell-wall-associated proteins and exoproteins. (Cheung et al., 2004). The sar locus encodes a DNA-binding protein SarA; a 14.7 kDa winged helix turn helix transcriptional activator and known to up-regulate the agr based quorum sensing system to elicit the exoprotein level (Cheung et al., 2004; Beenken et al., 2010). Simultaneously, the SarA indirect role on down-regulation of various other regulatory loci such as *rot, sarS, sarV, sarT* are well documented (Arya and Princy, 2013a). The DNA binding studies have revealed that the SarA binds to the intergenic region of P2 and P3 promoters of the *agr* locus and modulates the downstream genes such as *hla* and *spa* that encodes alpha hemolysin and protease respectively (Chien et al., 1999). But, the stable expression of both the genes in *agr* null strains strongly suggest the role of SarA on modulating its target gene expression either in direct or indirect manner (Cheung et al., 2008). Furthermore, SarA is also involved in the *agr*-independent expression of several other virulence genes including *fnbA* (fibronectin binding protein A), *TSS* (toxic shock syndrome) and *icaRA* (coagulase)*and bap* (biofilm associated proteins). Trotonda et al. (2005), Roberts et al. (2006), Andrey (2010) and Arciola et al. (2012). Existing antibacterial treatments for prosthetic vascular graft associated infections are inadequate due to the emergence of multi-drug resistance thus emphasing the need for combinatorial and higher dose therapy (Legout et al., 2014). So, there is a strong necessity for the novel therapeutic compounds to overcome antimicrobial resistance. In the current era few selective anti-virulent candidates have been revealed as a potent inhibitor of *S. aureus agr* based infections (Kiran et al., 2008; Sully et al., 2014). Targeting the SarA-DNA interaction has resulted in the identification of the first novel and extremely selective inhibitor that can efficiently suppress the staphylococcal infections. Hence, in an effort to develop an alternative treatment, several drug like molecules were designed and evaluated to assess their potentiality to overcome the pathogen responses to host tissues. Our study also demonstrates the advancement of research in this direction of exploiting the quorum regulator and score a novel potent SarA selective therapeutic candidate to dodge the *S. aureus* pathogenesis.

## Material and methods

### Bacterial isolates and growth conditions

*S. aureus* (105 strains) were isolated from rejected vascular graft (KAP Viswanatham Government Medical College, Trichy, India) and analyzed for the expression of virulence genes including antibiotic resistance, hemolysin production, biofilm formation and protease production. The strains that exhibited a higher degree of expression on those virulence factors were used for the drug efficacy analysis. To analyze the antimicrobial resistance pattern, the antibiotics such as penicillin, azithromycin, vancomycin, cefazolin, clindamycin, cloxacillin, erythromycin and teicoplanin were used in the study. The ATCC 25923 and mutant strains [Newman Δ*agr*::*tetM*, Newman *sarA*::Tn*917*LTV1, Newman Δ*agr*::*tetM, sarA*::Tn*917*LTV1] were grown aerobically at 37°C in tryptic soy broth (HiMedia, laboratories, India) overnight and the cultures were stored for further use (Table 1).

**Table 1:** **Bacterial strains used in this study**.

**Strain**	**Relevant genotype or phenotype**	**Reference/source**
ATCC 25923	clinical isolate	ATCC
ALC 355	Newman Δ*agr*::*tetM*	11
ALC 637	Newman, *sarA*::Tn*917*LTV1	11
ALC 638	Newman Δ*agr*::*tetM,sarA*::Tn*917*LTV1	11
QSLSA1051, QSLSA1052, QSLSA762, QSLSA764, QSLSA782, QSLSA785, QSLSA1061, QSLSA 1068, QSLSA 1149, QSLSA95, QSLSA1097	Wild type laboratory strain	This work

For broth culture, the *S. aureus* strains were grown in tryptic soy broth and the culture was incubated at 37°C with constant shaking at 200 rpm and the cells were harvested at exponential phase. The growth rate was measured spectrophotometrically at 600 nm (OD_600_). Wolz et al. (2000), Boles et al. (2010), Chen et al. (2011), Coraça-Huber et al. (2012).

### Computational methods

Structure assisted drug designing and molecular docking was used to design several drugs like molecules against the SarA DNA binding sites. Briefly, the 3-dimensional X-ray crystal structure of SarA was retrieved from the protein data bank [Accession ID: 2FRH] and further processed. The three amino acids D88, E89, and R90 responsible for conferring its binding to DNA (DNA binding domain) was selected as the target residues for the molecular docking analysis and the interactions of SarA with ligands formed the basis for design and scoring. Liu et al. (2006) and Arya and Princy (2013b).

The leads were subjected to the refinement as it improved their specificity, physiochemical properties including absorption, distribution, metabolism, excretion (ADME), and toxicity profile.

### Synthesis of SarA based biofilm inhibitor (SarABI)

The SarABI was synthesized by the condensation of commercially available 2,4 difluorobenzyl bromide and 4-aminocyclohexanol (Sigma-Aldrich) (Scheme-1).

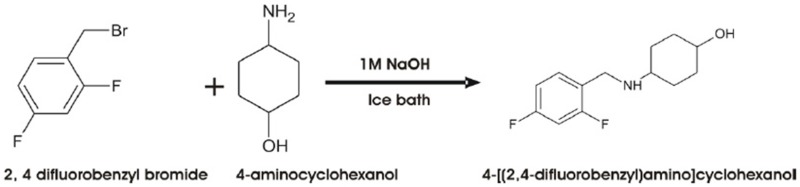



The condensation reaction was carried out on an ice bath in the presence of NaOH with constant stirring for 3 h. The reaction mixture was extracted with ethyl acetate. The pooled organic layers were washed successively and then washed with 10% HCl, 10% potassium carbonate and with brine, and dried over anhydrous magnesium sulfate. The formation of the SarABI was confirmed with proton and carbon nuclear magnetic resonance spectra followed by the gas chromatography mass spectra. The presence of hydroxyl group was confirmed by D_2_O exchange and Fourier transforms infrared spectroscopy (FT-IR using KBr).

### *in-vitro* analysis of SarABI efficacy

#### Minimum biofilm inhibitory concentration (MBIC)

A biofilm susceptibility assay (MBIC) was used to quantify the anti-biofilm activity of SarABI. *S. aureus* strains were grown overnight in TSB, diluted 1:100 in fresh TSB medium and grown to early exponential phase (OD_595_ = 0.2). Then 100 μl (approximately 2 × 10^7^ bacteria) of the culture were applied to sterile polystyrene 96-well plates along with 2-fold dilution series of SarABI and untreated culture was used as a control. The assay plates were incubated for 18 h at 37°C and then non-adherent cells were removed on repeating the washing step with phosphate buffered saline. The cells adhered to the polystyrene plates were stained with 50 μl of 0.06% crystal violet and the optical density was read at 600 nm to quantify the extent of biofilm formation. The concentration of the SarABI was used in a serial 2-fold dilution that could inhibit the biofilm formation by 50% (MBIC_50_) and 90% (MBIC_90_) compared to the untreated control (Opperman et al., 2012). The calculated MBIC_50_ and MBIC_90_ data were used in all the subsequent experiments to analyze the efficacy of SarABI.

#### Antimicrobial activity assay

*S. aureus* strains were grown overnight in TSB and diluted 1:100 in fresh TSB to reach early exponential phase of growth. Then 100 μl of this culture was applied to sterile 96-well polystyrene plates without or with the effective concentrations of SarABI (MBIC_50_ and MBIC_90_) was observed at 200 μg/ml and 1 mg/ml respectively). Cultures were grown without shaking for 24 h at 37°C and the optical density was measured at 595 nm, then 100 μl of culture were plated on to TSA for determining the colony forming units (Kiran et al., 2008).

#### Hemolysin production

Hemolytic activities of the SarABI were determined using rabbit erythrocytes. *S. aureus* were cultured overnight and diluted OD_600_ = 0.1 in 20 ml of fresh TSB and incubated for 3 h at 37°C till an approximate OD_600_of 0.6. The cells were collected by centrifugation at 10,000 × g for 10 min at 4°C and re-suspended in 20 ml of phosphate buffer saline (PBS). The 2% erythrocytes were prepared on centrifuging 1 ml of fresh de-fibrinated blood (1620 × g, 10 min) and the pelleted cells were re-suspended in 1 ml sterile PBS. The cells were repeatedly washed with PBS and re-suspended in 0.75 ml PBS and 2% erythrocyte suspension. Further 100 μl of the bacterial culture with or without SarABI (MBIC_50_ and MBIC_90_) as independent experiments were mixed with 900 μl of 2% red blood cells and incubated at 37°C for 3 h. The aliquot were centrifuged and the percent hemolysis was measured at an optical density of 540 nm (De Latour et al., 2010; Dean et al., 2011).

#### Bacterial cell adherence

##### Labeling *S. aureus* with fluorescein isothiocyanate (FITC)

The multi-drug resistant clinical isolates and reference strain were grown to early exponential phase. Cell pellets were re-suspended in PBS and sodium bicarbonate buffer with Fluorescein isothiocyanate (FITC) (1.0 mg/ml; Sigma Aldrich). The cells were incubated overnight at 4°C with gentle stirring and further washed with sodium bicarbonate buffer (Hochbaum et al., 2011).

##### Cell adherence

The FITC labeled *S. aureus* cells were diluted 1:100 in PBS. 100 μl of cells (2 × 10^7^) were applied to sterile cover slides in 12 well polystyrene plates with or without SarABI (at its effective concentration) and incubated for 30 min at 37°C. Thereafter cover slides were repeatedly washed with PBS and fluorescence determined at 485/530 nm (Zeiss AxioA1, Progress C5) (Bose et al., 2012).

##### Bacterial attachment to polystyrene *in vitro*

Briefly *S. aureus* strains were grown to the early exponential phase with OD_595_*nm* = 0.2 (contains 2 × 10^7^ bacteria). To analyze the cell attachment, 100 μl of the bacterial culture was taken in sterile 96 wells polystyrene plate with or without SarABI (at its effective concentration). Cells were grown for 3 h at 37°C and the unbound cells were removed on repeated washing with phosphate-buffered saline. The cells were air-dried and fixed with 100% ethanol and stained for 2 min with 0.4% gentian violet where the excess stains were removed on washing with PBS. A 100 μl of 1% sodium dodecyl sulfate (SDS) was added to each well to solubilize the stained cells. The optical density was measured at OD_595_ in 96 well plate reader (Biorad Plate Reader, Bio-Rad, Hercules, CA, USA) (Gov et al., 2001; Krut et al., 2003).

##### Confocal laser scanning microscope (CLSM) of static biofilm

Static biofilms were grown in a 8-well cover glass plates (Nunc, Wiesbaden, Germany) was subjected to analysis using confocal microscope (Olympus America, Inc., Melville, NY) after gentle washing and staining with fluorescent isothiocyanate (0.1% in PBS) for 15 min. The cells were washed twice in PBS and the developed biofilms were analyzed at excitation and emission wavelength 488 and 520 nm respectively by adjustable spectrum slit (Periasamy et al., 2012). Further the COMSTAT (Biofilm Image Processing Tool) was used to analyze the biofilm thickness, roughness, bio-volume and minimum colony size at the substratum.

#### Quantitative real time-PCR (qRT-PCR)

The total RNA was isolated from the SarABI (at its effective concentration) treated and untreated cells using guanidinium thiocyanate (Chomczynski and Sacchi, 2006).

The single step qPCR experiment was performed in a real-time cycler using SYBR Green method (Genotypic technology, India). The *fnbA, hld* and *RNAIII* expression patterns were determined using the following primers: 5′- TGCAAATACGACAGATACTT-3′(forward), 3′-TTGGCCACCTTCATAACCTA-5′ (reverse), 5′-ATGATCACAGAGATGGTA-3′(forward),3′-CTGAGTCCTAGGAAACTAACT-5′ (reverse),5′-CTGAGTCCTAGGAAACTAACTC-3′(forward),3′-TGATCACAGAGATGTGA-5′ (reverse). Relative levels (RL; %) of *fnbA, hld* and *RNAIII* transcripts were calculated using comparative Ct method and normalized to those of *arc* (carbamate kinase- house-keeping gene) transcript expression (Wolz et al., 2000).

### Rat graft *in-vivo* infection

The animal experiments were performed according to the experimental practices and standards developed by the animal welfare also with the prior approval from the Institutional Animal Ethics Committee (IAEC). Adult male Wistar rats (8–12 weeks) were used in the study, as it includes two series consisting of seven groups. Sterile collagen sealed double velour knitted polyethylene terephthalate (PET; Dacron) graft was used as the medical device in these experiments. For the animal experiments, the control group (disease control, DC) implanted with the unsoaked grafts and the experimental groups (infected either with the clinical isolates or the mutant strains) were implanted with SarABI soaked grafts as independent experiments were maintained. All rats were subjected to a minor surgery to make a subcutaneous pocket on each side of the median line by a 1.5 cm incision. All grafts were explanted after 15 days of implantation and biofilm formation was analyzed by determining the colony forming units. Various biochemical parameters such as albumin, ALT (alanine aminotransferase), ALP (alkaline phosphatase), AST (aspartate aminotransferase), direct bilirubin, total bilirubin, total protein, creatinine and urea were determined to analyse the toxicity of drug (Wang et al., 2011).

#### Histophalogy and bacterial count

Wistar rats were sacrificed after 15 days where the tissues from the graft site, liver, kidney, spleen, PET grafts and blood samples were collected. The tissue samples were further analyzed histopathlogically as well as processed for bacterial count. For histopathogical analysis, tissues were fixed in 10% formalin and sliced into a thickness of 2.1 mm. The tissues were then dehydrated with alcohol of graded concentrations. Subsequently the samples were cut on a microtome to 5 μm and stained with haematoxylin-eosin. The stained samples were examined under a light microscope; and photomicrographs of the samples were recorded (Bellows et al., 2011).

For bacterial count from various tissues, the samples were homogenized in PBS and serially diluted samples were then plated on 5% sheep blood agar plates. Similarly, 100 μl of blood samples were also cultured on blood agar plates. The collected PET grafts were sonicated in PBS so as to detach the adherent bacterial cells and the samples were cultured on blood agar plates to count bacterial cells (Aboshady et al., 2012).

### Cytotoxicity assay

The chemo-sensitivity of HEp-2 cells were determined using [3-(4, 5-dimethyl thiazol-2yl)-2, 5-diphenyl tetrazolium bromide] (MTT) standard assay. Briefly HEp-2 cell line (2 × 10^6^cells/ml) was seeded in 96-well plate. The adherent cells were grown to confluences for 24 h to allow cell attachment. The effective concentration of SarABI was added to each well and incubated for 72 h. The MTT solution was added and the cell viability was measured at 540 nm (George et al., 2012).

### Adherence assay to HEp-2 cell

Clinical isolates and mutant strains of *S. aureus* cells were labeled with fluorescein isothiocyanate (FITC) as described earlier (Balaban et al., 2003). To assess *S. aureus* adherence to HEp-2 human epithelial cells culture was applied to 8-well cover glass plates and allowed to grow at 37°C in a 5% CO_2_ incubator in bicarbonate-buffered dulbecco modified eagle medium (DMEM) (Sigma-Aldrich, USA) supplemented with 5% fetal calf serum (FCS) (Himedia. laboratories, India) to reach confluency (2 × 10^5^ cells/ml). FITC-labeled *S. aureus* (2 × 10^6^ cells/well in 90 μL PBS) were added to the confluent layer of HEp-2 cell line with or without SarABI at its effective concentration. *S. aureus* and HEp-2 cells were incubated for 30 min at 37°C and further washed with PBS and the intensity of fluorescence was determined at 485/530 nm under the CLSM.

### Statistical analysis

Statistical analysis was carried out using graph pad prism software (version 4.03). One-Way ANOVA was used, followed by Newman-Keuls multiple comparison test using GraphPad prism program version 6.0 (Graph Pad Software Inc., San Diego, CA). The minimum level of significance was set at *P* = 0.001. All assays were conducted in triplicates and statistical analysis was done.

## Results

### Design and synthesis of novel SarA based inhibitor (SarABI)

A pool of several hits was generated that could favorably interact to the DNA binding site of SarA using the *de novo* evolution mode of the program (Liu et al., 2006). The top-hit ligand was selected according to their relative energies, docking score, molecular and pharmacokinetic properties. The docking analysis revealed that the SarABI forms hydrogen bond with glutamic acid and arginine at 88 and 89 positions respectively. The reaction sequence leading to the formation of desired SarABI as outlined in scheme-1 yielded 72% of the compound. The presence of the hydroxyl group at characteristic position and number of protons (Figure S1) and carbons, the presence of benzene and cyclohexanol ring was confirmed (Figure S2). The D_2_O exchange and FTIR spectra confirmed the presence of amide and hydroxyl moiety in the SarABI (Figure S3). A corresponding molecular weight of 241 was confirmed with the GC-MS (Figure S4).

### Determine SarABI minimum biofilm inhibitory concentration (MBIC)

**Figure 1:**
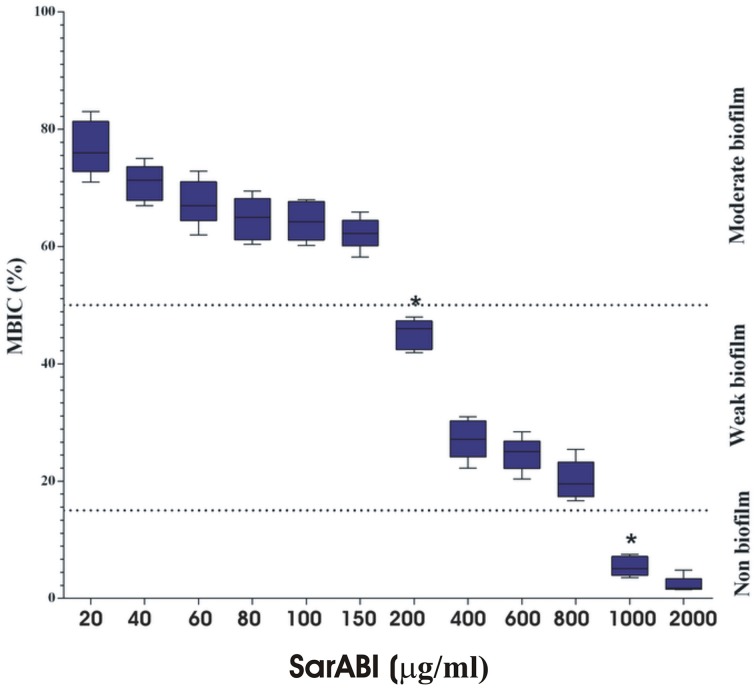
**Determination of minimum biofilm inhibitory concentration (MBIC)**. Cultures treated with various concentrations of SarABI were analyzed after 24 h comparing the effects of expressing a biofilm in *S. aureus* ATCC 25923. The static biofilm assay was performed with TSB medium, dotted line shown the difference between control and experimental groups. Statistical analysis was performed using the Two-Way ANOVA and Values of *P* < 0.001 were considered significant. Asterisk (^*^) indicates non detectable expression of biofilm.

The concentration of a drug required to either reduce 50% of biofilm deposition (MBIC_50_) or inhibit the biofilm formation up to 90% (MBIC_90_) was used as a standard for the assessment of SarABI sensitivities for multi-drug resistant*S. aureus*. The effect of SarABI on the biofilm formation at various concentrations were analyzed and it was observed that the reduction was prominent in a concentration-dependent manner. The SarABI exhibited an inhibitory effect on the development of *S. aureus* biofilm with MBIC_50_ of 200 μg/ml. No significant biofilm formation was observed in any of the clinical isolates when incubated with SarABI at a concentration of 1 mg/ml of the growth medium prior to inoculation (Figure 1).

### SarABI efficiently act on bacterial growth and hemolysin production

**Figure 2:**
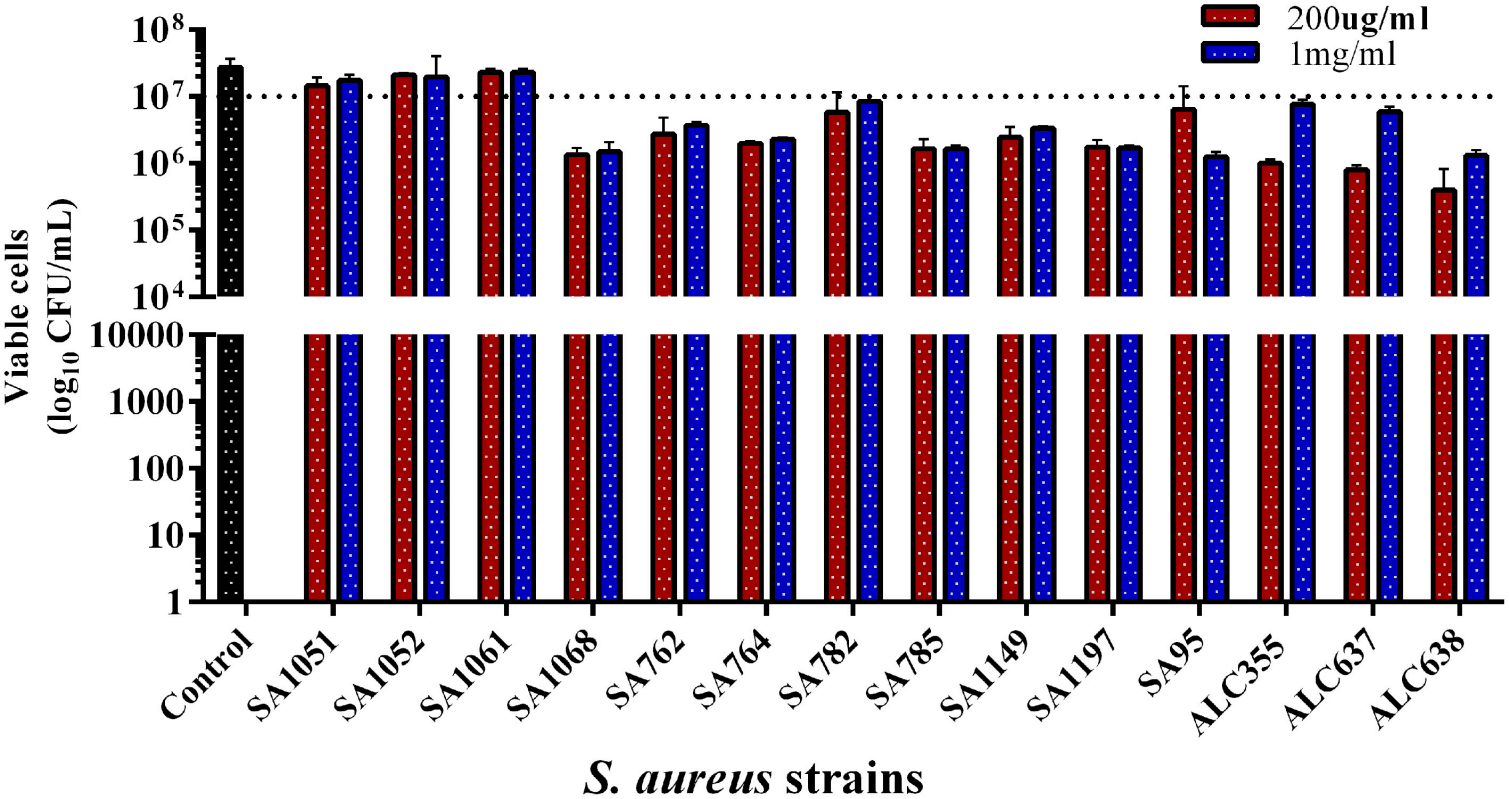
**Colony forming unit assays of clinical isolates and mutant strains of *S. aureus***. The strains were incubated for 24 h (2 × 10^7^ cfu/ml) with the addition of 200 μg/ml and 1 mg/ml SarABI in tryptic soy agar plates which does not affect the viability of clinical isolates compared to control. A similar effect was observed in mutant laboratory strains (ALC355, ALC637, ALC638), dotted line shown the difference between control and experimental groups. Statistical analysis was performed using the Two-Way ANOVA and Values of *P* < 0.001 were considered significant.

The drug, SarABI was designed to repress the expression of virulence genes, including biofilm formation and hence, did not exert any kind of selective pressure on bacteria to become resistant. The results obtained from colony forming units clearly indicated that SarABI did not exhibit antibacterial activity against *S. aureus*. The *in-vitro* antibacterial activity of SarABI with reference to its optimal concentration of MBIC_50_ (200 μg/ml) and MBIC_90_ (1 mg/ml) were tested on multiple drug resistant *S. aureus* strains. The study clearly indicated that the SarABI did not exhibit antibacterial activity against these strains up to the tested concentration of 1 mg/ml (Figure 2).

**Figure 3:**
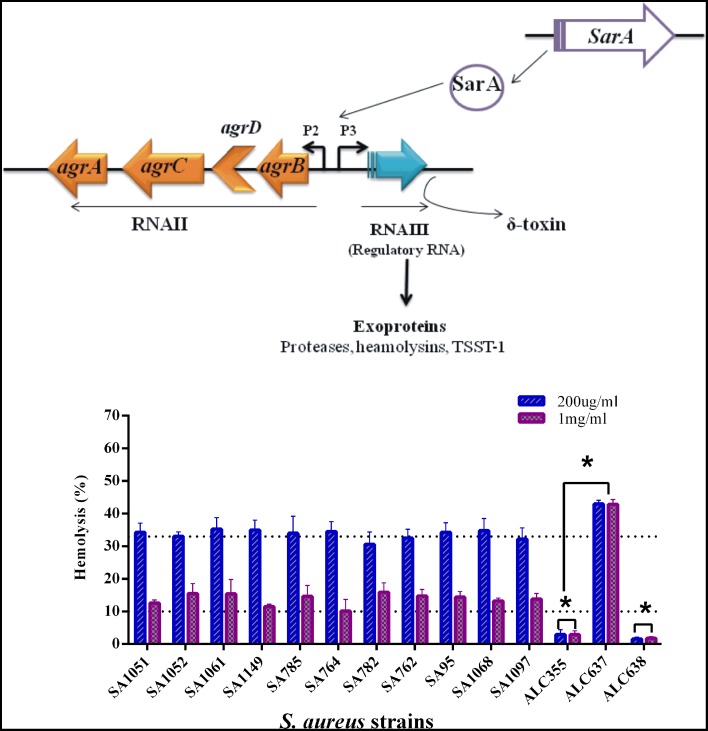
**Synergistic in comparison to direct lysis of rabbit blood erythrocytes by *S. aureus w*ith SarABI**. Culture supernatants were grown in presence (200 μg/ml and 1 mg/ml, respectively) or absence of SarABI, culture filtered and incubated with a 2% solution of defibrinated rabbit blood for 30 min at 37°C. Hemolysis was measured by determining OD_540_nm *using* spectrophotometer and % lysis calculated from lysed erythrocyte standards, dotted line shown the difference between control and experimental groups. Statistical analysis was performed using the Two-Way ANOVA and Values of *P* < 0.001 were considered significant. SarABI significantly decreases the expression of hemolysin in all experimental groups. Asterisk (^*^) indicates no weak expression of hemolysin.

As shown in Figure 3, hemolysin activity was significantly decreased in the clinical isolates and *agr* mutant strain (Newman Δ*agr*::*tetM*) when treated with SarABI but considerably increased in the *sarA* mutant (Newman *sarA*::Tn*917*LTV1) as compared to the control. These results are consistent with the data that the SarABI shows SarA selective suppression of the biofilm formation. The hemolytic activity of the clinical isolates and mutant strains were also tested and it was found that 200 μg/ml of SarABI had reduced the hemolytic activity to 50% and >100% was observed as the drug (SarABI) dose was increased to 1 mg/ml.

### Small molecule SarABI, attenuate the bacterial cell adherence

**Figure 4:**
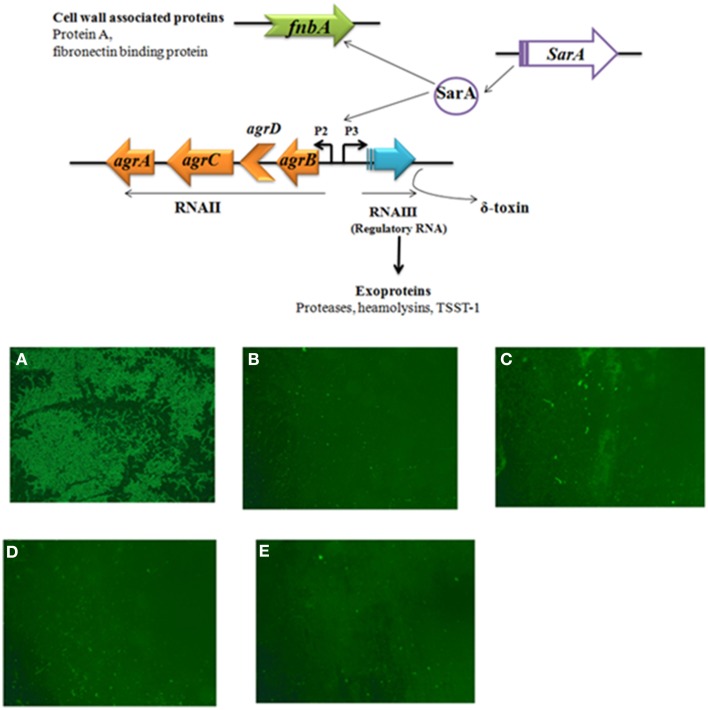
**Effective concentration of SarABI reduced for the adherence of *Staphylococcus aureus*. (A)** FITC labeled ATCC 25923 cells (10^9^ CFU) were applied to glass slide with 100 μl of PBS, **(B)** clinical isolate SA1061, **(C)** ALC637 (*sarA*::Tn*917*LTV1), **(D)** ALC355 (Δ*agr*::*tetM*), **(E)** ALC638 (Δ*agr*::*tetM sarA*::Tn*917*LTV1) effective concentration of SarABI were used for the adherence. Cells were incubated for 24 h at 37°C, unbound cells were removed by PBS washing and adherent cells observed under the fluorescent microscopy 5x.

The study was performed to analyze whether the results from extracellular protein secretion and biofilm formation have precisely mirrored the bacterial attachment. So, the *S. aureus* strains listed in Table 1 were compared for their cell adherence capacity on the solid surface. The *sarA* mutant (Newman *sarA*::Tn*917*LTV1) and *agr sarA* double mutant (Newman Δ*agr*::*tetM, sarA*::Tn*917*LTV1) strains were observed to be weak biofilm produces and naturally lost their ability to adhere to the solid (polystyrene plate) surface. As expected, the SarABI treatment caused a remarkably reduced adherence in all clinical strains along with the *agr* mutant under static conditions subjected to an incubation for 30 min (Figure 4). The data clearly implies the major role of SarA played a major role in the activation of key *S*. *aureus* surface associated genes.

#### SarABI significantly inhibit biofilm formation

**Figure 5:**
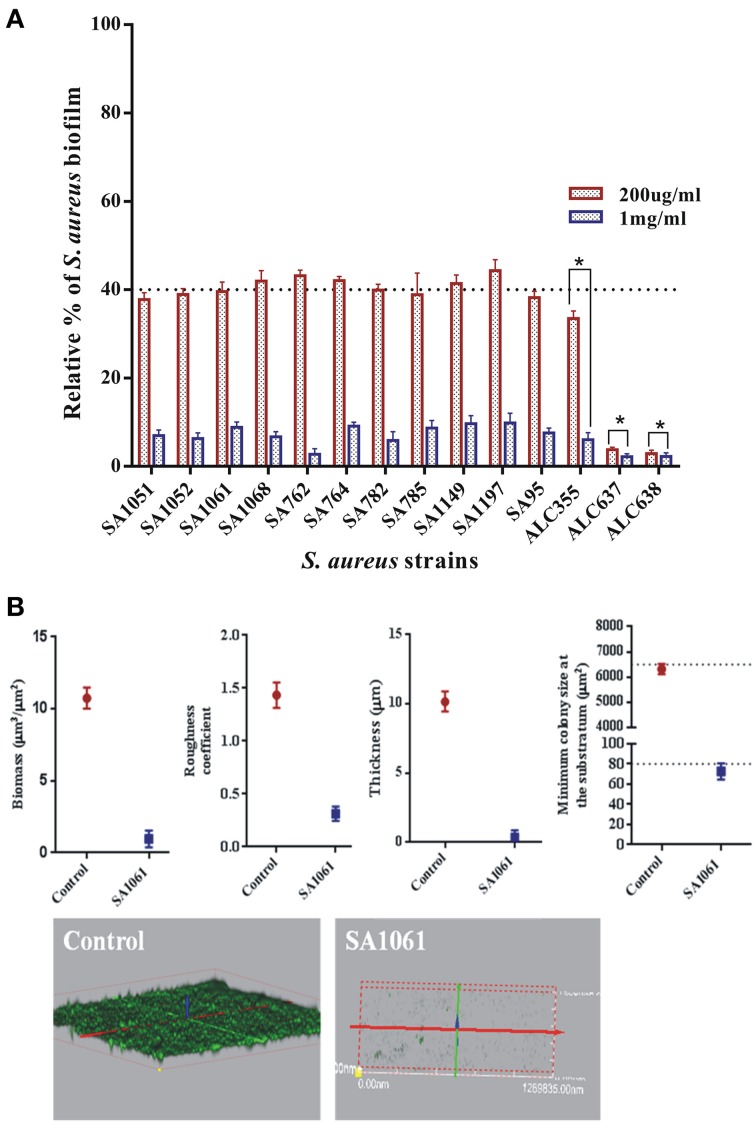
**(A)** The effect of SarABI on *S. aureus* attachment to polystyrene *In vitro*: Cell attachment compared with the clinical isolates and the mutant backgrounds ALC355 (Δ*agr::tetM*), ALC637 (Δ*sarA::Tn917LTV1*) single mutants, and the ALC638 (Δ*agr::tetM*, Δ*sar::Tn917LTV1* double mutants) treated with SarABI. *S. aureus* cultures grown in 96-well microtiter plates were pretreated with SarABI (200 μg/ml and 1 mg/ml respectively) and incubated for 24 h. Values show mean numbers of biofilm formation/well, and error bars indicate range, dotted line shown the difference between control and experimental groups. Statistical analysis was performed using the Two-Way ANOVA and values of *P* < 0.001 were considered significant. SarABI significantly decreases the expression of biofilm in all experimental groups. Asterisk (^*^) indicates no detectable expression of biofilm. **(B)** Quantitative analysis of biofilm development on coverslips by *S. aureus* strains treated with SarABI using CLSM shows biomass, roughness coefficient, thickness and minimal colony at the surface and biofilm production by *S. aureus* as assessed by three dimensional images compared with untreated biofilms. Statistical analysis was performed using the Two-Way ANOVA and values of *P* < 0.001 were considered significant. Asterisk (^*^) indicates drastically decline fluorescent intensity.

The efficacy of SarABI on biofilm inhibition was studied *in situ* over time for all the strains using the confocal laser scanning microscopy (CLSM). As shown in Figure 5, the real time measurement of biofilm thickness using time lapse CLSM imaging revealed that untreated clinical isolate, SA1061 has shown a significantly higher biofilm formation with a thickness up to 11 μm in elevation over the surface. On the other hand, the SarABI treatment affected the biofilm forming ability of the *S. aureus* clinical isolates, ATCC 25923 and the *agr* mutant strains SA1061 strain displayed the highest level of total biomass (11 μm^3^/μm^2^) and maximum thickness (>10 μm). In contrast, a sharp reduction in biovolume ranging from 0.2 to 1.9 μm^3^/μm^2^ and thickness (0.2–1.0 μm) was observed in all the treated strains. Similarly, a fivefold reduction in the roughness coefficient was observed in SarABI treated strains (~0.3 μm^3^/μm^2^) than that of control (1.4 μm^3^/μm^2^). Minimum colony size at the substratum for treated *S. aureus* strains ranged from 50 to 75 μm^2^ as compared to that of control where a considerable larger colony size (6000 μm^2^) was observed. Prominently, these results emphasize that the SarA was not a sole regulator to control the biofilm formation as earlier presented but also epitomized as a crucial molecular factors contributing to biofilm structuring in *S. aureus*.

### SarABI targets SarA based major virulence genes expression

**Figure 6:**
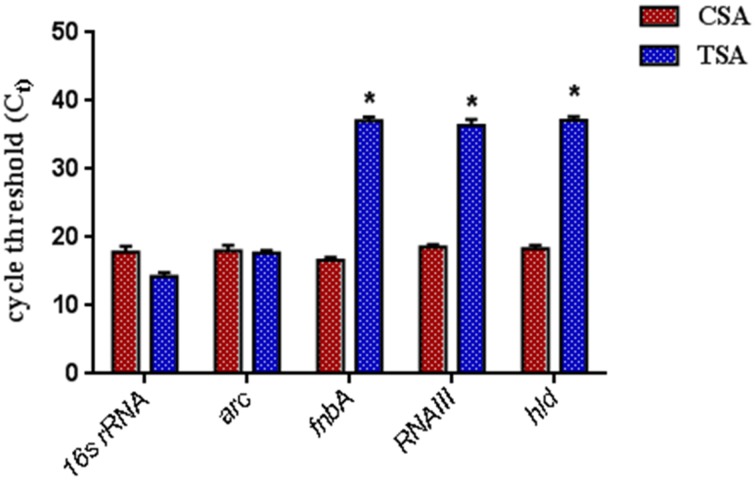
**The validation of virulence gene expression level using quantitative RT-PCR**. *S. aureus* were grown overnight at 37°C with (TSA, treated S. *auerus*) or without (CSA, control *S. aureus*) treated of 1 mg/ml SarABI in tryptic soy broth. Expression pattern of the *fnbA, hld*, and *RNAIII* were diminished in cells treated with SarABI in comparison to without treated. Statistical analysis was performed using the Two-Way ANOVA and values of *P* < 0.001 were considered significant. Asterisk (^*^) indicates significantly low expression of genes.

The SarABI, an inhibitor suppressed the transcriptional activator SarA and negatively controlled it to down-regulate its target genes that influences the adhesion molecules biosynthesis as well as certain toxin production. Hence, inactivation of temporal expression of these genes should invariably affect the expression of RNAIII, δ-hemolysin (*hld*) and fibronectin binding protein A (*fnbA*). To determine the fact whether the SarA selective inhibitor (SarABI) has down-regulated the phenotype, biofilm and other virulence factors, the quantitative real time PCR (qPCR) analysis of their transcript levels were also done. As shown in Figure 6, the treatment of highly virulent clinical isolate, SA1061 with SarABI (TSA) at a concentration of 1 mg/ml greatly reduces the expression of *fnbA, RNAIII*, and *hld* as compared to the untreated (CSA) strain. The expression of house-keeping genes, *arc* and *16s rRNA* were also quantified to analyze the effect of SarABI on the survival of *S. aureus*. No significant changes in the expression of those reference genes were observed in both TSA and CSA. Hence the results were consistent to show the SarABI, a well-characterized SarA based inhibitor.

### SarABI treatment *in-vivo* diminishes vascular graft infection

**Figure 7:**
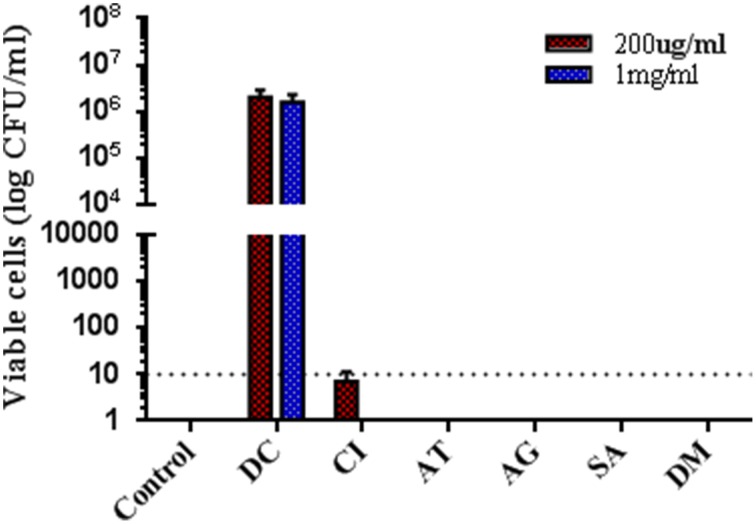
**Quantitative microbiological evaluation experiments shows SarABI reduced *in-vivo* infection**. Bacteria (2 × 10^7^CFUs) were incubated with SarABI for challenge the animals. After 14 day incubation, the graft was removed, and the number of bacteria were determined on blood agar plates. The graph depicts group-I (control), group-II (DC, Diseased control infected with ATCC 25923), group-III (CI, *S. aureus clinical* isolate with SarABI), group-IV (AT, ATCC 25923 with SarABI), group-V (AG, *agr::tetM w*ith SarABI), group-VI (SA, *sar::Tn917LTV1* with SarABI) and group-VII (DM, *agr::tetM, sar::Tn917LTV1* with SarABI). The low and effective doses of SarABI were used for studied, dotted line shown the difference between control and experimental groups. Statistical analysis was performed using the One-Way ANOVA and values of *P* < 0.001 were considered significant. Asterisk (^*^) indicates no detectable bacteria, suggesting <10 CFUs/ml.

The SarA selective inhibitor, SarABI has proven to show anti-adherence effect to potentiate its role to overcome the device-associated infections *in-vivo*. Graft presoaked with 200 μg/ml and 1 mg/ml showed no sign of infection even though the animals were challenged with a high bacterial load of 2 × 10^7^ cfu and were analyzed by the colony forming units on blood agar plates (Figure 7).

**Figure 8:**
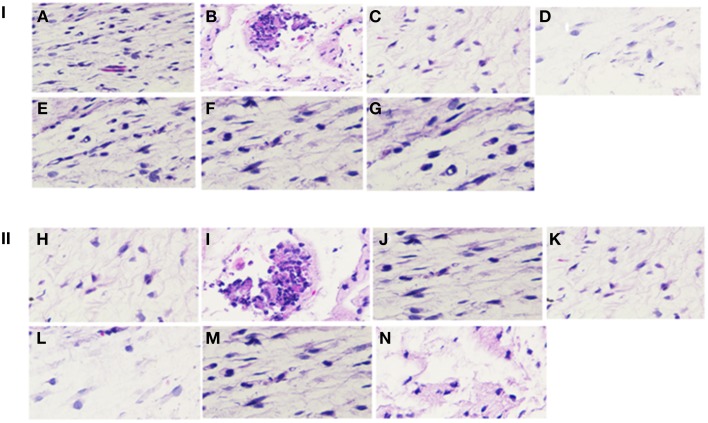
**Hematoxylene and eosin stained sections of tissues from implant site**. Skin ulcer and necrotic debris was observed in disease control group. Disease control group (B, I) also exhibits a marked tissue reaction with predominant macrophages, fibroblasts, lymphocytes and neutrophils along with collagen deposition. A fibrous capsule and granulation tissue formation with angiogenesis was predominantly present in the sections. **(I,II)** represents the 200 μg/ml and 1 mg/ml SarABI coated vascular grafts experimental groups respectively. Representative samples from different groups treated with SarABI do not show any significant difference from saline control (A, H), DC (B, I), CI (C, J), AT (D, K), ALC355 (E, L), ALC637 (F, M) and ALC638 (G, N).

Biochemical analysis of the serum sample was performed to evaluate the SarABI toxicity effect over the function of the renal and the hepatitis systems. The data showed that the SarABI at its effective concentrations (200 μg/ml and 1 mg/ml) inhibits the *S. aureus* biofilm and virulence but did not affect the normal function of the host cells in comparison with the control (untreated groups) (data not shown). Similarly, histopathological studies of SarABI effect on wistar rats demonstrated that even administration of higher dosage of this drug does not cause any change in the cellular integration of the liver, kidney, spleen along with the epithelial cells from the graft site while the lesion was observed in adjacent to the epithelial cells from the graft site of the untreated group. All the organs from the drug treated group were found within the histological limits as compared with the control group (Figure 8).

### Cell proliferation not affected by SarABI

The cytotoxic effect of the effective concentration of SarABI (200 μg/ml and 1 mg/m) on the cell viability were evaluated. A compound usually is considered to have *in vitro* cytotoxic effect if the particular concentration of drug caused a 50% cell death. In this study, the cell viability was found to be higher than 95% in all the drug doses and hence, the SarABI showed no cytotoxic effect.

### SarABI reduces adherence of *S. aureus* on Hep-2 cells

**Figure 9:**
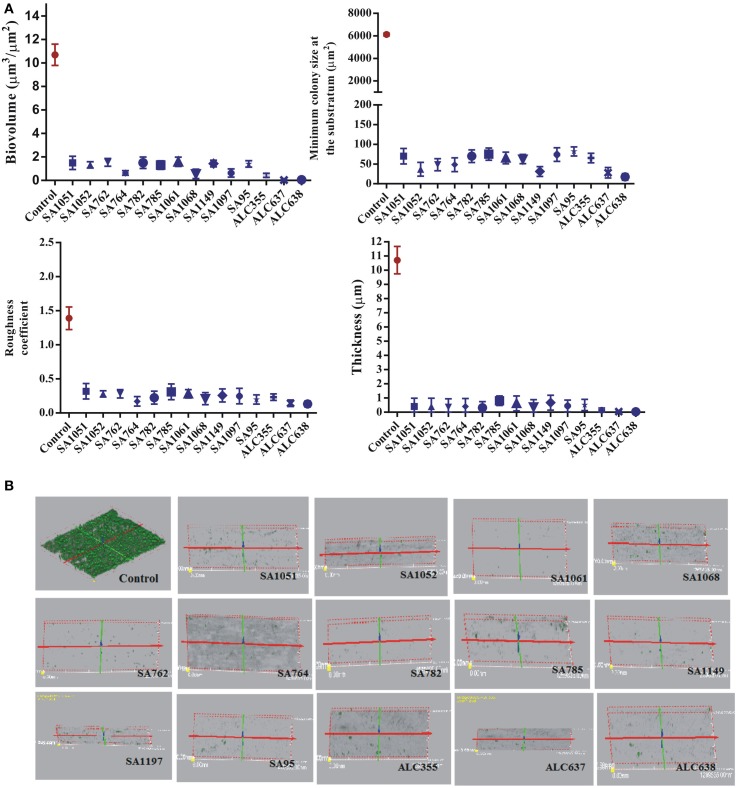
**Effect of SarABI on adherence of *S. aureus* to HEp-2 cell lines**. The graph depicts Control (with normal saline), DC (Diseased control contaminated with ATCC 25923), CI (*S. aureus* clinical isolate with SarABI), AT (ATCC 25923 with SarABI), ALC355 (*agr::tetM w*ith SarABI), ALC637 (*sar::Tn917LTV1* with SarABI) and ALC638 (*agr::tetM, sar::Tn917LTV1* with SarABI). The effective concentration of SarABI synergistically diminished the adherence of *S. aureus* to HEp-2 cell lines. **(A)** Data revealed the significantly reduce in biomass, roughness coefficient thickness and colony at substrate compared with untreated samples. **(B)** Three-dimensional reconstructed renderings of the *S. aureus* adherence to HEp-2 cells on coverslip. The effective doses of SarABI were used for studied. Statistical analysis was performed using the Two-Way ANOVA and values of *P* < 0.001 were considered significant. Asterisk (^*^) indicates significant result.

To investigate the role of SarABI on preventing the S. *aureus* from colonizing the confluent layer of Hep-2 cells, a control (ATCC 25923), clinical isolate and three mutant strains were subjected to CLSM analysis and further the influence of drug (SarABI) over its adherence influenced by various parameters (function of bio volume, roughness coefficient, colony size and mean thickness) were characterized using COMSTAT. The data critically revealed a differential pattern of adherence between the treated and untreated groups to Hep-2 cells under the same conditions other than the mutant strains (Newman *sarA*::Tn*917*LTV1, Newman Δ*agr*::*tetM* and *sarA*::Tn*917*LTV)., As shown in the Figure 9, the untreated control produces a biovolume of 11 μm^3^/μm^2^ while the SarABI treatment at a concentration of 1 mg/ml significantly reduced the biovolume ranging from 0 to 2 μm^3^/μm^2^. The SarABI treatment also reduced the biovolume in the strain, ALC355 while ALC638 did not exhibit any biofilm formation. The minimum colony size at substratum analysis showed that the SarABI treatment reduced the colony size from 6000 μm^2^ (untreated) to 50 μm^2^. The roughness coefficient was also reduced from 1.4 to nearly 0.3 for all the clinical isolates when treated with SarABI. A higher biofilm was measured in the control and exhibited 11 μm thickness in comparison with the treated isolates and the thickness of the biofilm ranged from 0 to 1 μm. Also the influence of the drug (SarABI) to inhibit the *S. aureus* adherence to the host cell is consistently similar to the effect achieved from our previous adherence assay carried out on the glass surface.

## Discussion

The study demonstrates a unique approach to inhibit the staphylococcal virulence and pathogenesis via., inhibiting the SarA based quorum sensing. The method demonstrated the inhibition of virulence gene expression rather than killing the bacteria. In our previous study, we have used *denovo* computer-aided discovery of novel SarA selective inhibitors against the target, SarA (PDB ID: **2FRH**) (Arya and Princy, 2013b). The SarABI has shown better interaction with the protein, SarA by H-bond acceptor and donor, presence of aromatic rings, functional groups and hydrophobic sites. These interactions provided the SarA-SarABI to form a stable complex as it laid a key role to negatively regulate the SarA interaction to its target promoter regions. The complex was also stabilized by hydrogen bond interactions with the Asp and Glu residues at 88 and 89th position in SarA and ensures to bind to the hydrophobic clamp. Also the presence of fluorine, hydroxyl and amine group might have an imperative role in the inhibition activity.

The compound, SarABI was further tested for its potency at inhibiting biofilm formation against all clinical isolates and isogenic (*agr* and *sarA*) mutant strains. The *agr* null strain had shown a significant higher expression of biofilm while the expression was reduced when treated with SarABI. The result suggested that the SarABI affects the biofilm on negatively regulating SarA-specific interaction of SarABI to downregulate SarA targeted genes expression that establishes biofilm. Furthermore, the cell adherence assay also revealed that the untreated clinical isolates, *agr* null strain and the reference strain, ATCC 25923 exhibited a higher adherence on the glass surface while the SarABI treatment inhibited the bacterial cells attachment. The *sarA* and *agr sarA* double mutant strains (Newman Δ*agr*::*tetM, sarA*::Tn*917*LTV1) either treated with SarABI or untreated did not induce biofilm formation. These results confirmed that SarABI acts via opposing the effects of SarA and most likely inhibited the binding of SarA to the DNA (Arya and Princy, 2013b) hence affecting the quorum sensing processes. Thus, the SarA selective inhibitor, SarABI was found to be very effective in suppression of the process of cell attachment, proliferation and invasion.

The biofilm formation in *S. aureus* is a multistep process that commences with the cell attachment and then the expression of genes responsible for extracellular toxins production (Lister and Horswill, 2014). An array of hemolytic proteins is frequently isolated from *S. aureus* and is among the most significant staphylococcal toxins. The hemolysins and associated proteins are pore-forming staphylococcal virulence factors that significantly contribute in bacterial infections (Tavares et al., 2014). A concentration-dependent inhibition of hemolytic activity was observed when the clinical isolates were treated with SarABI.

As the effective concentration of 200 μg/ml (MBIC_50_) and high dose of 1 mg/ml (MBIC_90_) of SarABI were found to inhibit the biofilm formation as observed in the MBIC assay, we furthermore sought to define and analyze the impact of the drug on the biofilm formation using fluorescence labeling of the biofilm. There was substantial reduction in the degree and kinetics of biofilm formation with the strains treated with SarABI. In static biofilms, biovolume, roughness coefficient, colony size and mean thickness values were considerably higher in the ATCC 25923, *agr* mutant, Newman Δ*agr*::*tetM* and all clinical isolates compared with those values of the treated strains.

The expression of the various genes including RNAIII*, fnbA* and *hld* plays an imperative role in staphylococcal pathogenesis including biofilm formation, proliferation and evasion (Beenken et al., 2010). The transcription of RNAIII was greatly reduced in the *S. aureus* culture supplemented with SarABI and hence changed the temporal expression of various virulence factors. The lack of adherence and biofilm formation in *sarA* mutant (Newman *sarA*::Tn*917*LTV1) and SarABI treated clinical isolates also suggested that the activation of fibronectin-binding proteins promotes their adherence to the surface or host epithelial cells is under the direct control of SarA. This was remarkably substantiated by the RT-qPCR data where the down-regulation of all those genes was detected during exponential phase after treatment, demonstrating SarABI interference with SarA to show its response to its target genes at the transcriptional level. As expected, the expressions of all three transcripts were correlated with the decreased biofilm formation and hemolysin activity of all clinical strains.

The cytotoxicity of the SarABI was also evaluated and results demonstrated that the SarABI did not show cytotoxic effect to HepG-2 cell line even at its higher concentrations. Remarkably, SarABI a novel drug to show its effect on inhibiting a quorum regulator, SarA and further to downregulate several gene expression that establishes biofilm and virulence without affecting the cell growth. So, this approach would block an independent pathway other than the vital pathways responsible for their life cycle. The approach toward finding an “anti-virulent” drug in the control of pathogenic bacteria imposes a control over its pathogenic phenotypes rather than developing selective pressure and drug resistance.

## Author contributions

All the authors have equally contributed to the manuscript.

### Conflict of interest statement

The authors declare that the research was conducted in the absence of any commercial or financial relationships that could be construed as a potential conflict of interest.

## Supplementary material

Figure S1 **Chemical characterization of the Trans-4-[(2, 4-difluorobenzyl) amino]cyclohexanol (SarABI) after an 8 h reaction with catalyst NaOH**. The distribution of characteristic aromatic and aliphatic protons according to their occurrence in those regions was confirmed with proton NMR. The hump region denotes the presence of NH group.

Figure S2 **^13^C NMR Spectra of SarABI**. The carbon NMR spectrum was used to confirm the two benzene rings along with the diamine aliphatic region according to their prediction space in the spectra.

Figure S3 **Characteristic FTIR absorbencies of the SarABI**. Major vibrational bands interpreted to be due to: H-bonded OH-stretching (3415.42 cm^−1^).

Figure S4 **Mass Spectroscopy Determination of SarABI was analyzed by GC/MS in CI mode using Helium flow for ionization to determine molecular weights**. The sample was pyrolysed at 290°C, and the resulting vapor was injected into gas chromatograph mass spectrometer (GC–MS). The different gaseous products were separated by the GC, and analyzed by MS in the GC column outlet and identify the products by their mass spectrum.
