# Supplementary figures and images for: SarA based novel therapeutic candidate against Staphylococcus aureus associated with vascular graft infections
Source: Front Microbiol. 2015 May 6;6:416. doi: 10.3389/fmicb.2015.00416 (PMC4447123; doi:10.3389/fmicb.2015.00416)

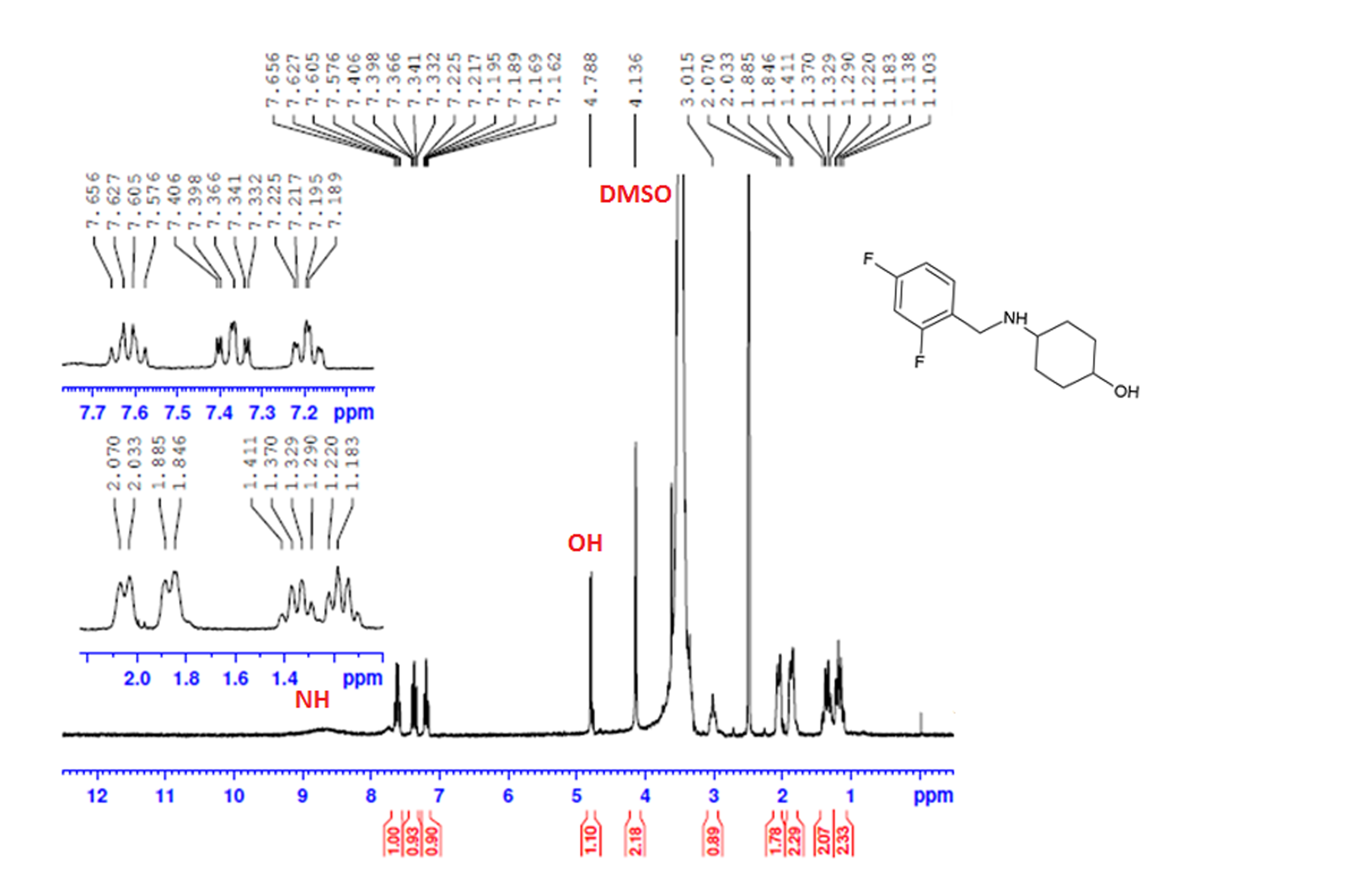

Supplement: Figure S1 — Chemical characterization of the Trans-4-[(2, 4-difluorobenzyl) amino]cyclohexanol (SarABI) after an 8 h reaction with catalyst NaOH. The distribution of characteristic aromatic and aliphatic protons according to their occurrence in those regions was confirmed with proton NMR. The hump region denotes the presence of NH group. [file Image1.TIF]

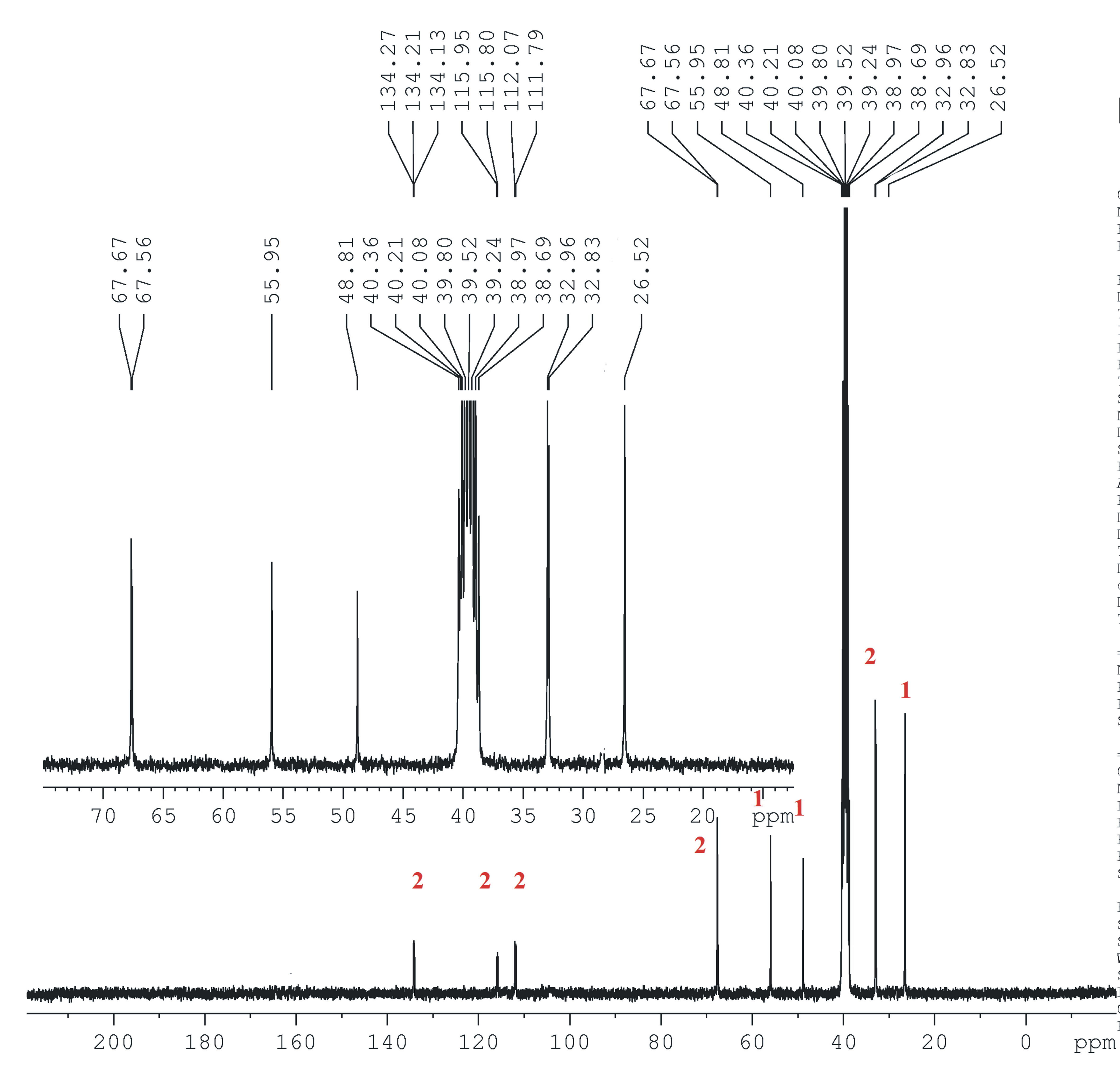

Supplement: Figure S2 — 13C NMR Spectra of SarABI. The carbon NMR spectrum was used to confirm the two benzene rings along with the diamine aliphatic region according to their prediction space in the spectra. [file Image2.TIF]

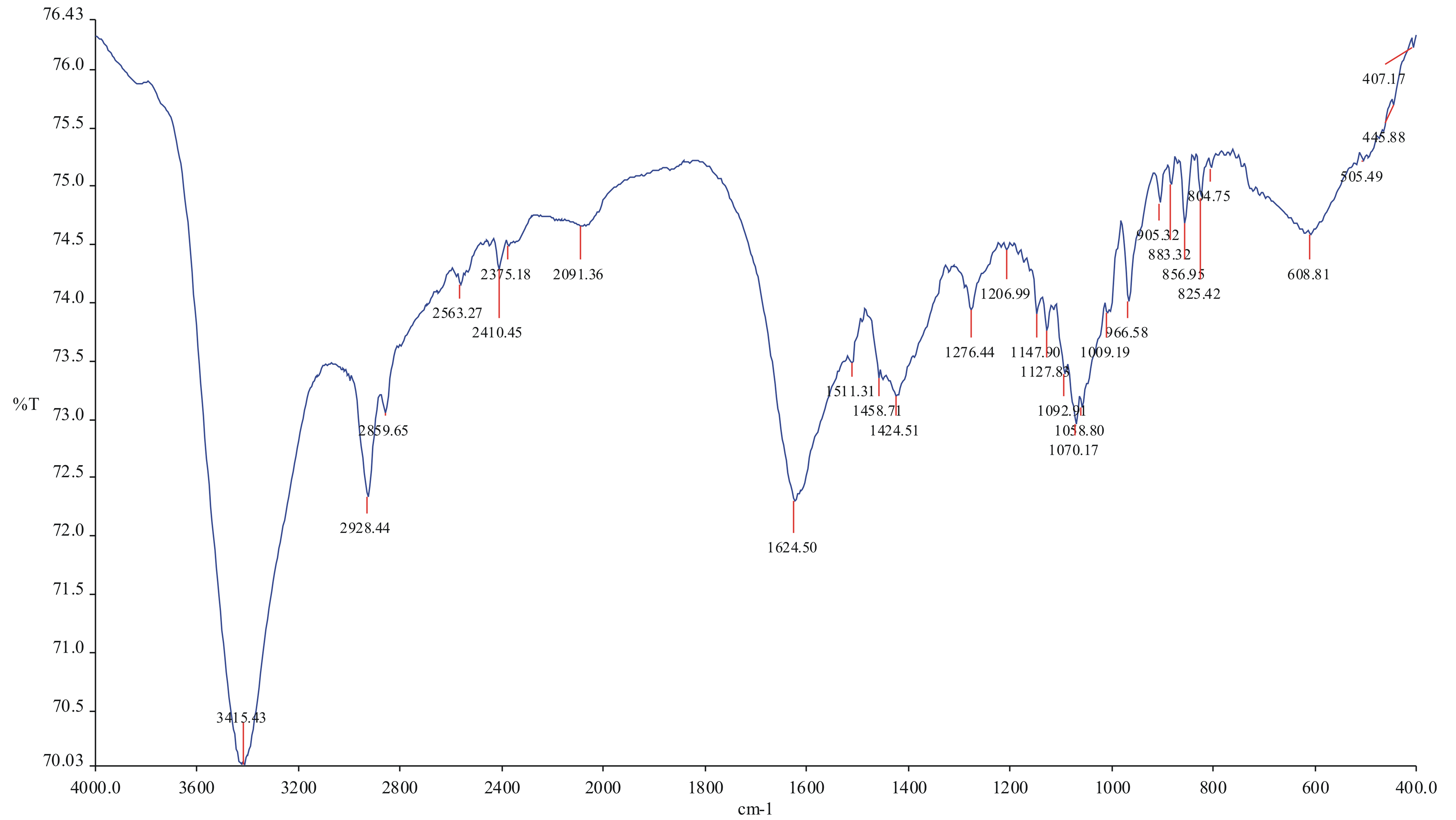

Supplement: Figure S3 — Characteristic FTIR absorbencies of the SarABI. Major vibrational bands interpreted to be due to: H-bonded OH-stretching (3415.42 cm−1). [file Image3.TIF]

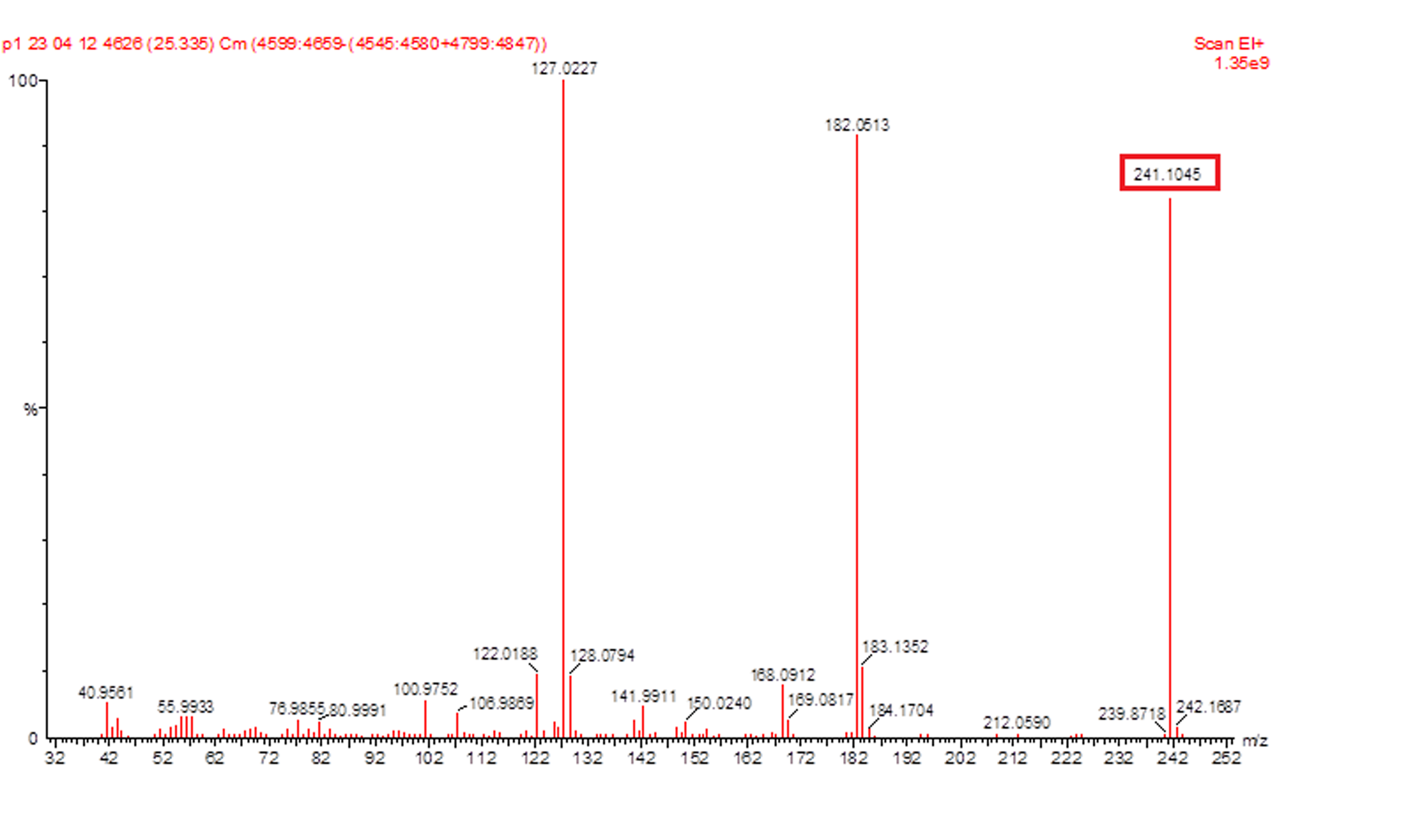

Supplement: Figure S4 — Mass Spectroscopy Determination of SarABI was analyzed by GC/MS in CI mode using Helium flow for ionization to determine molecular weights. The sample was pyrolysed at 290°C, and the resulting vapor was injected into gas chromatograph mass spectrometer (GC–MS). The different gaseous products were separated by the GC, and analyzed by MS in the GC column outlet and identify the products by their mass spectrum. [file Image4.TIF]
