# Supplementary material for: SarA based novel therapeutic candidate against Staphylococcus aureus associated with vascular graft infections
Source: Front Microbiol. 2015 May 6;6:416. doi: 10.3389/fmicb.2015.00416 (PMC4447123; doi:10.3389/fmicb.2015.00416)
Supplement: Supplementary file 5 [file Table1.DOCX]

**Table S1. Activity of selected antibiotics against *Staphylococcus aureus* isolates. The activity were depicted as Resistant (**+**), Susceptible (**-**) and intermediate (**+/-**).**

| **Strain Name** | **Antibiotic susceptibility test** | | | | | | | | | | | | |
| --- | --- | --- | --- | --- | --- | --- | --- | --- | --- | --- | --- | --- | --- |
|  | **Pen** | **Azi** | | **Van** | **Cfz** | **Cd** | **Clx** | **Ery** | **Tei** | **Ofx** | **Gen** | **Ctz** | **Cf** |
| SA95 | + | | - | - | +/- | - | - | + | - | +/- | - | - | - |
| SA1051 | + | | - | +/- | - | - | - | +/- | +/- | + | - | - | +/- |
| SA1052 | + | | - | +/- | +/- | - | - | +/- | +/- | - | - | - | - |
| **SA1061** | **+** | | **-** | **+/-** | **+/-** | **-** | **-** | **+/-** | **+/-** | **+** | **+** | **+/-** | **+/-** |
| SA1149 | + | | - | - | +/- | - | - | - | - | +/- | - | - | +/- |
| SA1097 | + | | - | - | - | - | +/- | +/- | - | - | - | +/- | +/- |
| SA1068 | + | | - | - | - | - | +/- | - | - | +/- | - | - | + |
| SA762 | + | | - | - | - | - | +/- | - | - | + | +/- | + | + |
| SA785 | + | | - | +/- | - | - | - | - | - | + | +/- | + | + |
| SA764 | + | | - | - | - | - | - | +/- | - | + | + | +/- | +/- |
| SA782 | + | | - | - | - | - | - | - | - | + | + | + | - |

**Abbreviations:** Pen-Penicillin, Azi-Azithromycin, Van-Vancomycin, Cfz-Cefazolin, Cd- Clindamycin, Clx- Cloxacillin, Ery- Erythromycin, Tei- Teicoplanin. Ofx-Ofloxacin, Gen-Gentamicin, Ery-Erythromycin, Ctz-Clotrimazole, Cf- Cephalothin.
