# Supplementary material for: SarA based novel therapeutic candidate against Staphylococcus aureus associated with vascular graft infections
Source: Front Microbiol. 2015 May 6;6:416. doi: 10.3389/fmicb.2015.00416 (PMC4447123; doi:10.3389/fmicb.2015.00416)
Supplement: Supplementary file 6 [file Table2.DOCX]

**Table S2. Expression of hemolysin production in *Staphylococcus aureus* isolates.**

| **Strain Name** | **Hemolysin production** | | |
| --- | --- | --- | --- |
|  | **Alpha** | **beta** | **Gamma** |
| SA95 | + | + | + |
| SA1051 | + | + | - |
| SA1052 | + | - | - |
| **SA1061** | **_+_** | **+** | **+** |
| SA1149 | + | + | - |
| SA1097 | + | - | + |
| SA1068 | + | + | - |
| SA762 | + | - | - |
| SA785 | + | - | + |
| SA764 | + | + | - |
| SA782 | + | + | - |

**Abbreviations: + symbol depicts the presence of hemolysin, - symbol depicts the absence of hemolysin.**
