# Supplementary material for: SarA based novel therapeutic candidate against Staphylococcus aureus associated with vascular graft infections
Source: Front Microbiol. 2015 May 6;6:416. doi: 10.3389/fmicb.2015.00416 (PMC4447123; doi:10.3389/fmicb.2015.00416)
Supplement: Supplementary file 7 [file Table3.DOCX]

**Table S3. Expression of protease produced by *Staphylococcus aureus* isolates.**

| **Strain Name** | **Protease production** |
| --- | --- |
| SA95 | + |
| SA1051 | + |
| SA1052 | ++ |
| **SA1061** | **_+++_** |
| SA1149 | +++ |
| SA1097 | ++ |
| SA1068 | ++ |
| SA762 | ++ |
| SA785 | ++ |
| SA764 | ++ |
| SA782 | + |

**Abbreviations: The proteolysis results shows as in the diameter produced on agar plates supplemented with 5% NaCl and 1% casein. +symbol shows 15-20mm diameter, ++symbol shows the 20-25mm diameter and +++ symbol shows the >25mm diameter.**
